# Visceral leishmaniasis mimicking aggressive lymphoma: Concurrent *Mycobacterium africanum* lymphadenitis in an immunocompetent adult

**DOI:** 10.1016/j.idcr.2026.e02819

**Published:** 2026-10-28

**Authors:** Nahid Ahmed, Justin Low, Richard Hinton, Brendan O’Kelly

**Affiliations:** a Department of Infectious Diseases, Our Lady of Lourdes Hospital, Drogheda, Ireland; b Royal College of Surgeons in Ireland, 123 St Sptehen's Green , Dublin 2, D02YN77, Ireland; c Department of Haematology, Our Lady of Lourdes Hospital, Drogheda, Ireland

**Keywords:** Visceral leishmaniasis, Leishmania donovani complex, Mycobacterium africanum, Lymphoma mimic, Lymphadenitis, Pancytopenia, Diagnostic anchoring, Liposomal amphotericin B

## Abstract

Visceral leishmaniasis (VL) may be an important lymphoma mimic because it may produce a similar presentation, including prolonged fever, significant weight loss, massive splenomegaly, cytopenias, diffuse lymphadenopathy, and bone marrow abnormalities (Eddine, 2026).This report presents a case of an immunocompetent 45-year-old man with prior residence in West Africa and prolonged residence in Spain who presented with fever, night sweats, gastrointestinal symptoms, abdominal pain, splenomegaly, lymphadenopathy, and pancytopenia. Initial work-up included a cervical lymph node biopsy, which demonstrated necrotising granulomas; Xpert MTB/RIF was positive, and the culture identified Mycobacterium africanum. Antituberculous therapy was initiated, but the patient developed hepatotoxicity and did not improve clinically. Specialist testing of existing admission bone marrow specimen detected Leishmania donovani complex DNA by polymerase chain reaction. Liposomal amphotericin B was administered at 4 mg/kg on days 1–5, 10, 17, 24, 31, and 38 (cumulative dose 40 mg/kg). This exceeded the commonly used 20 mg/kg total dose for immunocompetent VL (Bern et al., 2006) and was selected as an individualized multidisciplinary decision in the setting of concurrent tuberculosis lymphadenitis and recent high-dose prednisolone exposure after marrow sampling. The patient had marked clinical and laboratory improvement. This case may illustrate concurrent infections presenting as suspected lymphoma and may highlight the diagnostic value of reassessing stored tissue when a confirmed diagnosis does not explain the entire phenotype.

Clinical significance

A microbiologically accurate initial diagnosis may result in premature diagnostic closure [1]. In this case, persistent fever, marked splenomegaly, and cytopenias may not have been fully explained by proven *M. africanum* lymphadenitis alone; the lack of clinical response to standard antituberculous treatment further supports the possibility of a concurrent process.

## Introduction

Visceral leishmaniasis, a potentially fatal systemic infection if left untreated, and is caused predominantly by the Leishmania donovani complex [2]. Its clinical manifestations—fever, weight loss, splenomegaly, cytopenias, hyperferritinaemia and lymphadenopathy—overlap substantially with haematological disorders such as lymphoma [3]. Corticosteroid-treated patients have been reported with disseminated or diagnostically delayed leishmaniasis [4]; in the present case, however, the brief three-day steroid exposure is presented as a reason for diagnostic vigilance, not as evidence that steroids worsened the infection; this interpretation reflects a multidisciplinary team’s clinical judgment, and the literature does not appear to provide a validated duration threshold. Premature lymphoma-directed therapy should likewise be regarded as a potential diagnostic hazard when infection has not been excluded, rather than as a proven cause of adverse outcome in this patient.

The diagnostic challenge may be amplified when another infection is microbiologically proven. Tuberculosis and leishmaniasis may coexist [5], and a positive mycobacterial test may create diagnostic closure if persistent constitutional symptoms, splenomegaly or cytopenias are attributed to a single process [6]. An earlier report specifically describes VL as initially misdiagnosed as malignant lymphoma [7], while a recent case report again emphasised the lymphoma-like presentation of VL [3]. We describe concurrent *M. africanum* lymphadenitis and VL in an immunocompetent adult, with diagnosis established by retrospective molecular testing of stored marrow.

## Case presentation

A 45-year-old immunocompetent man presented on 5 November 2025 with fever, rigors, night sweats, significant weight loss, anorexia, loose stools and left-sided abdominal pain. He was born in Nigeria, moved to Ghana in early childhood, lived in Spain for more than two decades and had moved to Ireland two years before presentation. He travelled frequently to Spain and Ghana, including travel to Spain from July to early August 2025. He reported no recognised sandfly or animal exposure, known tuberculosis contact, or significant previous medical history.

On examination, the patient was febrile (>38 °C) and had marked splenomegaly and cervical lymphadenopathy. Initial investigations showed a white-cell count of 2.6 × 10⁹/L, neutrophils 1.82 × 10⁹/L, lymphocytes 0.55 × 10⁹/L, haemoglobin 11.4 g/dL and platelets 121 × 10⁹/L. C-reactive protein was 48 mg/L, ferritin 995 μg/L and lactate dehydrogenase 677 U/L; renal function was normal. Chest radiography showed no pulmonary focus. Computed tomography (CT) demonstrated small supraclavicular lymph nodes, a heterogeneous 16-cm spleen and diffuse retroperitoneal adenopathy, with clear lungs (Fig. 1, Fig. 2, Fig. 3).

**Fig. 1:**
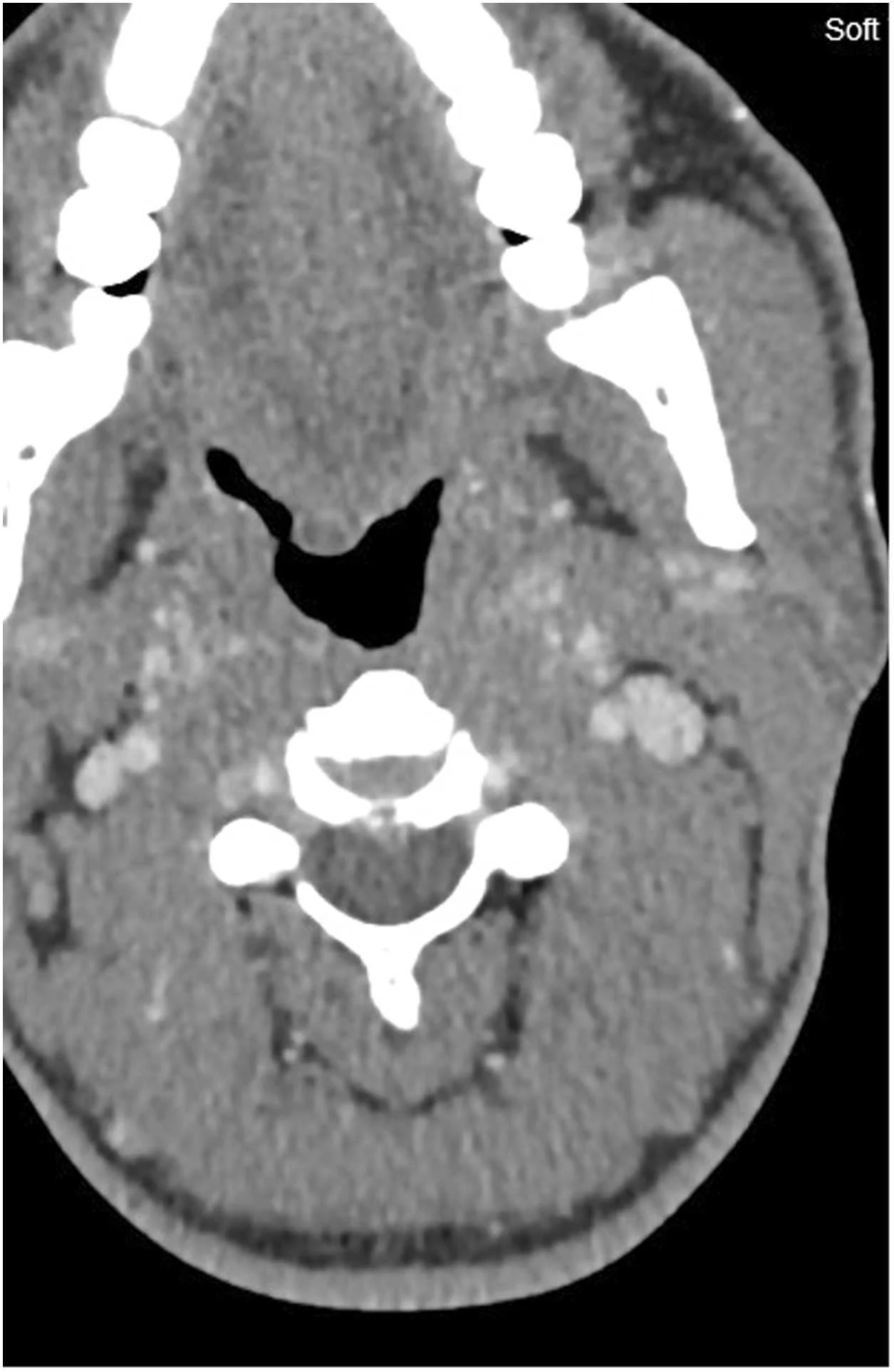
Axial contrast-enhanced CT image through the upper neck demonstrating small cervical and supraclavicular lymph nodes in the setting of suspected lymphoma.

**Fig. 2:**
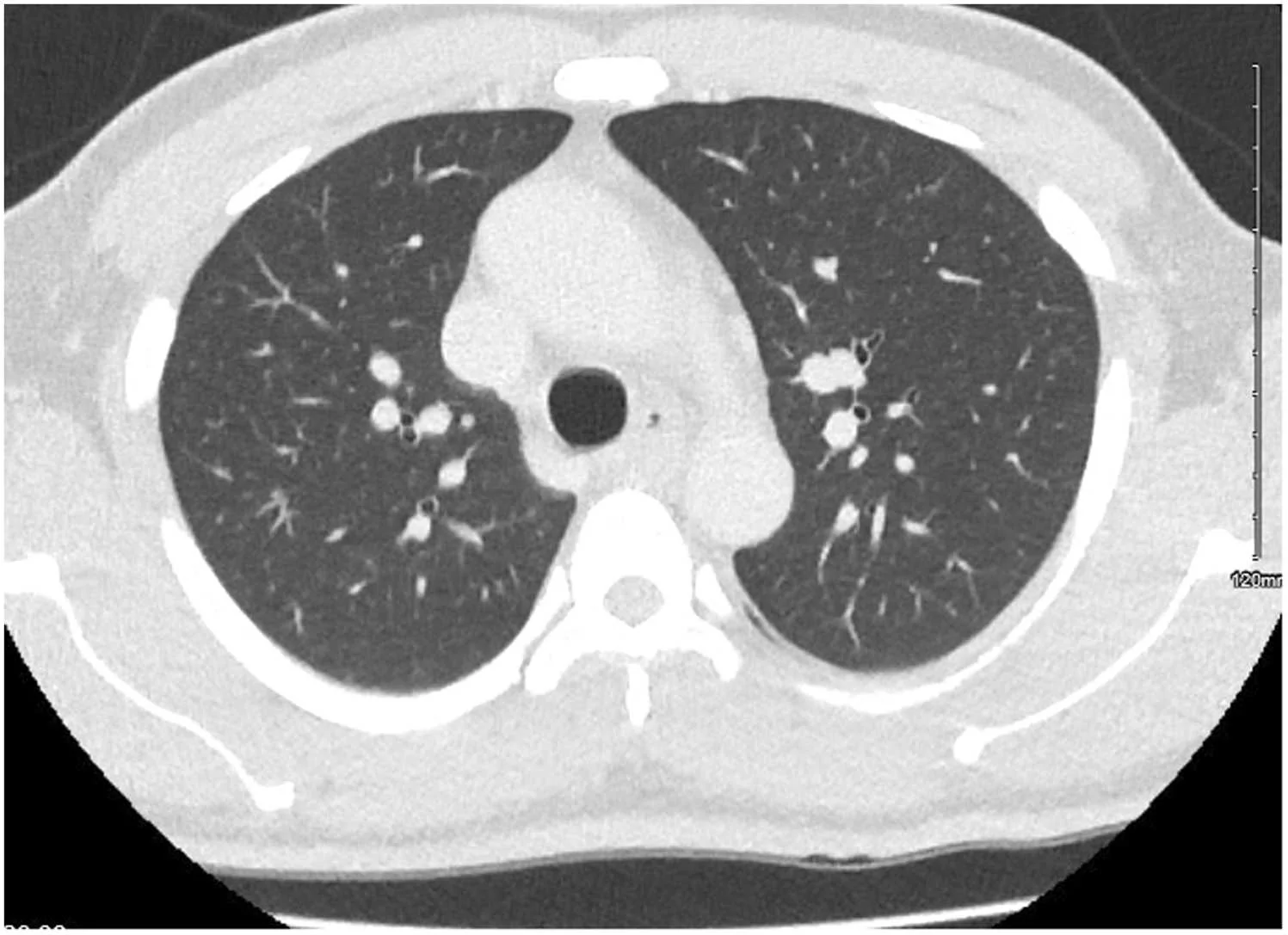
Axial CT image of the thorax in a lung window, showing clear lungs without a focal pulmonary lesion or pleural effusion.

**Fig. 3:**
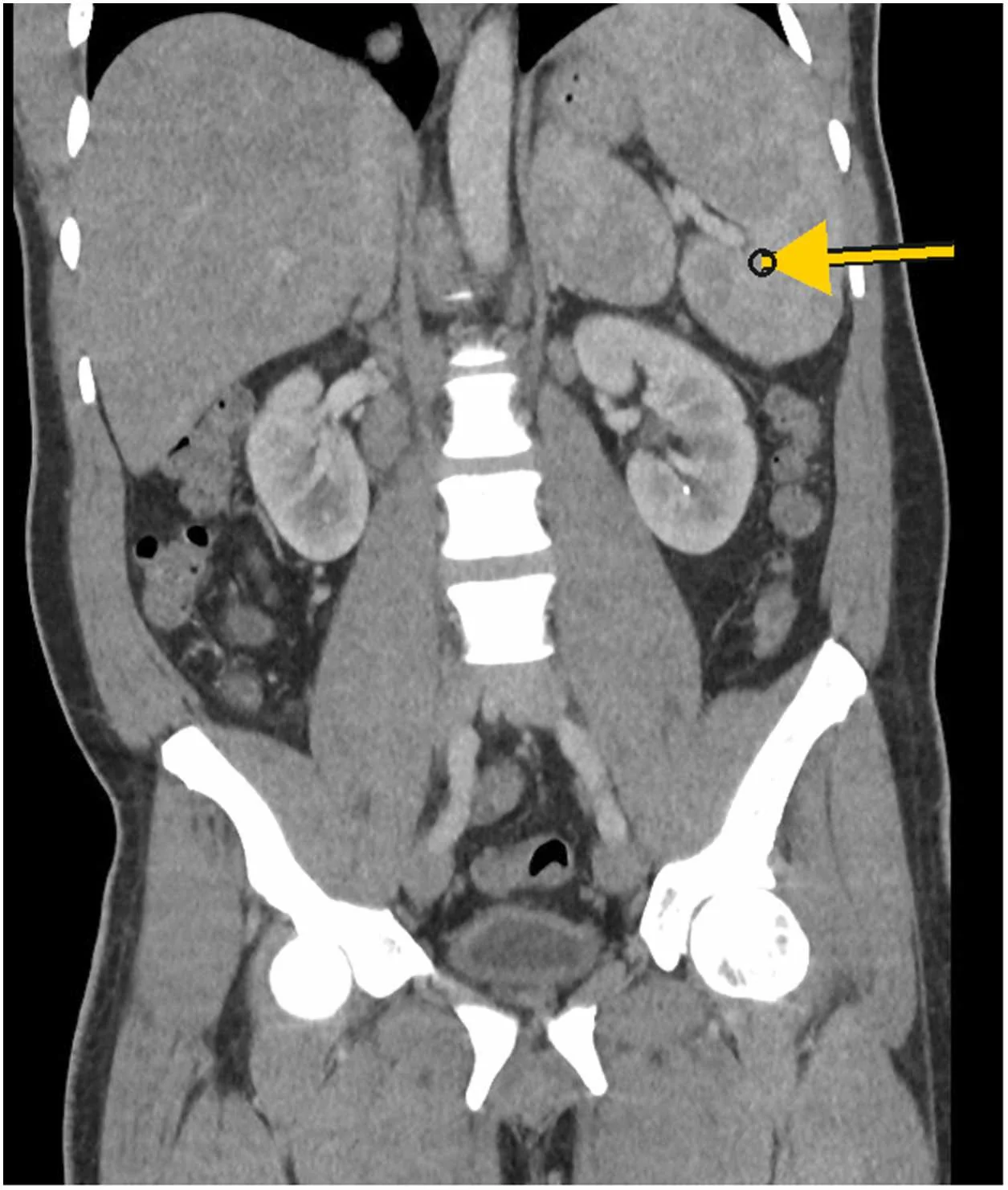
Coronal CT image of the abdomen and pelvis demonstrating marked splenomegaly and associated retroperitoneal lymphadenopathy.

### Initial lymphoma and tuberculosis work-up

Because the combination of constitutional symptoms, splenomegaly, cytopenias and lymphadenopathy strongly suggested lymphoma, bone marrow aspirate and trephine biopsy were obtained on 5 November 2025. Prednisolone 80 mg orally once daily was started after marrow sampling but was discontinued after three days when the preliminary marrow assessment showed no evidence of lymphoma.

A cervical lymph node biopsy demonstrated necrotising granulomas. Xpert MTB/RIF detected Mycobacterium tuberculosis complex, and culture subsequently identified Mycobacterium africanum susceptible to rifampicin, isoniazid, pyrazinamide and ethambutol, but resistant to streptomycin. Antituberculous therapy was started on 13 November 2025.

### Hepatotoxicity and persistent diagnostic discordance

On 19 November, alanine aminotransferase was 334 U/L, aspartate aminotransferase 583 U/L, and bilirubin 33 μmol/L. Antituberculous drugs were withheld. After biochemical improvement, treatment was reintroduced sequentially from 25 November: rifampicin, then isoniazid and ethambutol, with levofloxacin replacing pyrazinamide. The temporal association with drug exposure, improvement after withdrawal, and tolerance of sequential reintroduction appeared to support antituberculous drug-induced liver injury, although hepatic involvement from VL remained a competing explanation [8].

Despite microbiologically appropriate antituberculous therapy, the patient’s fever, splenomegaly, cytopenias, and constitutional symptoms persisted, raising concern for a concurrent infection or underlying malignancy. A reference laboratory subsequently reported PCR detection of Leishmania donovani complex in the bone marrow specimen obtained early in the admission. This result prompted initiation of treatment for visceral leishmaniasis, which was considered a concurrent infection contributing to the persistent clinical features.

### Diagnosis and treatment of visceral leishmaniasis

The Parasitology Department at University College London tested the pre-existing bone-marrow specimen. Leishmania donovani complex polymerase-chain-reaction testing was positive on 28 November 2025. Retrospective review of the bony marrow biopsy did reveal macrophage rich marrow with suspicious intracellular inclusions ( Fig. 4, Fig. 5). After multidisciplinary discussion, liposomal amphotericin B was administered at 4 mg/kg on days 1–5, 10, 17, 24, 31 and 38 (10 doses; cumulative dose 40 mg/kg). A total dose of approximately 20 mg/kg is generally supported for an immunocompetent patient with visceral leishmaniasis (VL) [9]. Therefore, 40 mg/kg was not intended as the standard regimen for an immunocompetent host. It was an individualized institutional decision, made after specialist and multidisciplinary discussion, because this patient had severe disseminated disease, concurrent M. africanum lymphadenitis and several days of prednisolone 80 mg orally once daily given after marrow sampling for presumed aggressive lymphoma. The short steroid course was considered a clinically relevant transient immunomodulating exposure and a reason to favour a cautious treatment strategy. The dosing decision may therefore be regarded as precautionary. The patient’s substantial clinical improvement was observed following the initial 5 doses of VL treatment. Post-treatment monitoring revealed the normalisation of haematological indices and the resolution of hepatosplenomegaly, which may indicate a favourable therapeutic response.

**Fig. 4:**
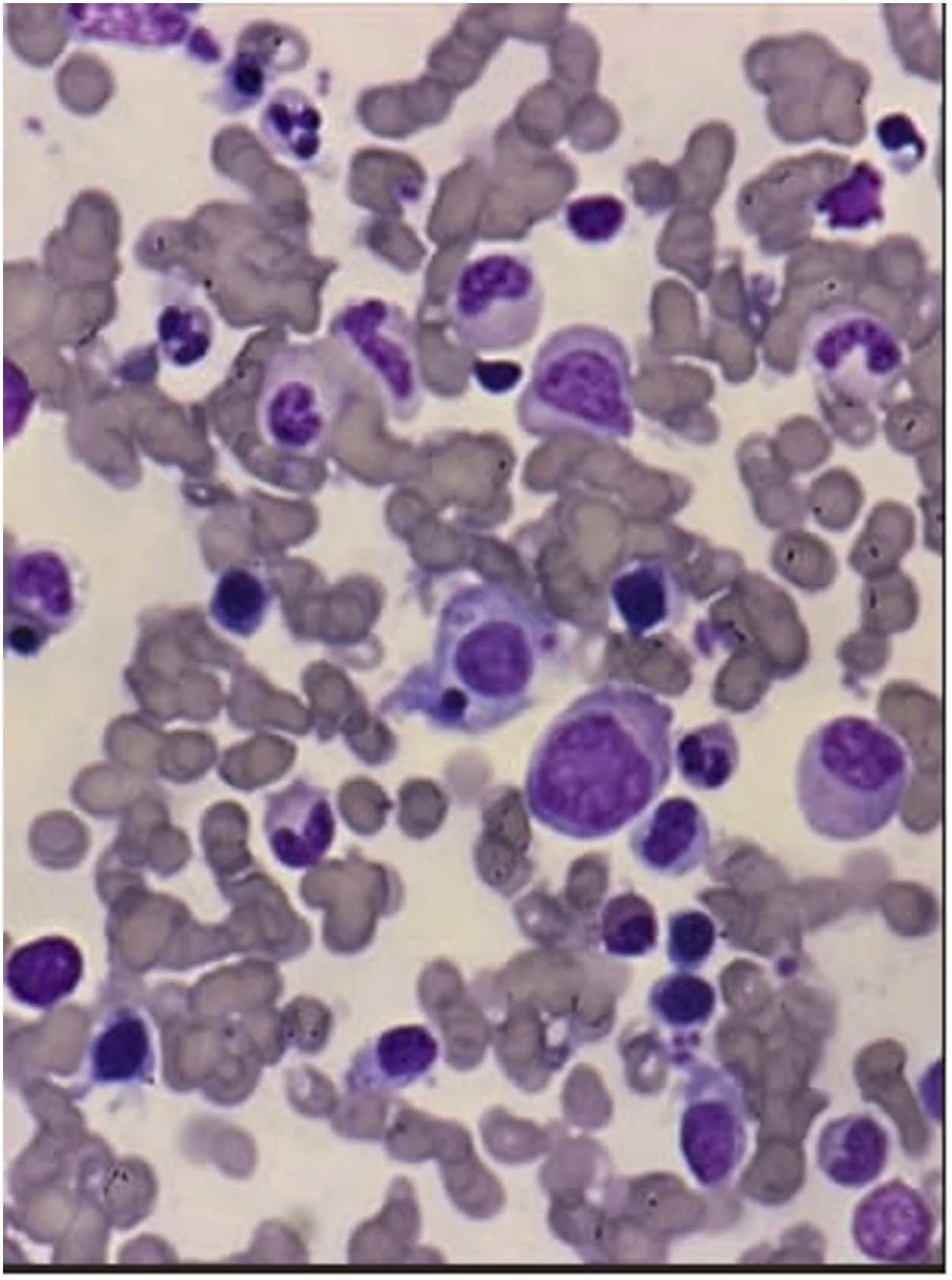
Bone-marrow field showing macrophage-rich marrow with intracellular inclusions that prompted specialist review.

**Fig. 5:**
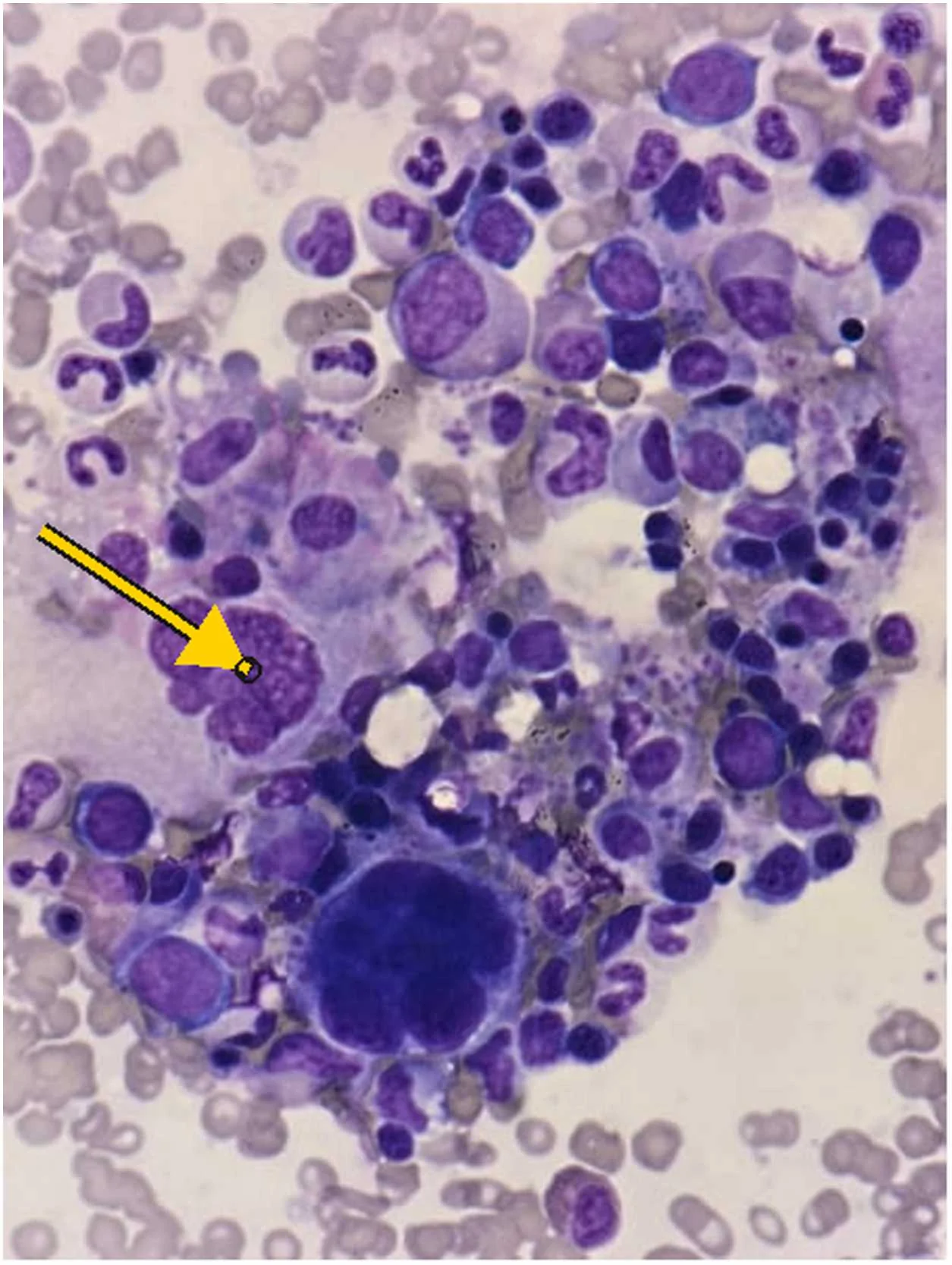
Additional bone-marrow image showing a cellular haematopoitic population with macrophages and suspicious inclusion bodies.The arrow identifies the highlighted inclusion body for specialist review.

The patient was discharged on 10 December 2025. He completed outpatient tuberculosis treatment on 2 July 2026. Monthly follow-up documented resolution of fever and abdominal symptoms, normalisation of blood counts and recovery of liver biochemical parameters.

## Timeline

Date

Clinical event

5 Nov 2025

Admission with fever, weight loss, splenomegaly, lymphadenopathy and cytopenias; bone marrow sample obtained by the haematology team.

5–7 Nov 2025

Prednisolone 80 mg/day started after marrow sampling and stopped when lymphoma was not seen.

13 Nov 2025

Antituberculous therapy started after necrotising granulomas and positive Xpert MTB/RIF from cervical node.

19 Nov 2025

Hepatotoxicity detected; antituberculous drugs withheld.

25 Nov 2025

Sequential reintroduction of rifampicin, isoniazid and ethambutol; levofloxacin substituted for pyrazinamide.

28 Nov 2025

Stored marrow tested positive for the Leishmania donovani complex by polymerase chain reaction (PCR).

Nov–Dec 2025

Liposomal amphotericin B administered; clinical improvement and discharge on 10 Dec.

2 Jul 2026

Tuberculosis treatment completed; blood counts, liver tests and clinical findings normal.

## Discussion

This case suggests that a proven infection may be real but incomplete as an explanation for the overall syndrome. VL may closely mimic lymphoma: fever, weight loss, splenomegaly, cytopenias, elevated LDH, and marrow abnormalities can be produced by either disease [10]. The diagnosis in this patient was further obscured by simultaneous M. africanum lymphadenitis, a plausible explanation for necrotising granulomas and a positive molecular test.

The travel history supported epidemiological plausibility without identifying the exact time or site of acquisition. M. africanum is strongly associated with West Africa [11], while L. infantum and L. donovani complex transmission occurs across parts of the Mediterranean basin, Africa and Asia [12]. The absence of a recalled sandfly bite does not necessarily exclude VL, and clinical history should be interpreted alongside tissue findings and targeted molecular testing.

Following bone marrow sampling, prednisolone 80 mg orally once daily was administered for three days because aggressive lymphoma was initially suspected. It was promptly discontinued when lymphoma was not demonstrated. It is reported here as a transient immunomodulating factor that justified heightened diagnostic vigilance in a patient with active tuberculosis and persistent systemic features. Importantly, marrow morphology raised suspicion but PCR appears to have provided the decisive evidence.

The hepatic injury was temporally associated with antituberculous therapy and improved after drug withdrawal, supporting drug-induced liver injury. Nevertheless, the two processes may coexist. The practical approach is therefore not to force a single attribution but to reassess the entire clinical picture, review stored tissue with specialist laboratories and test for a second treatable infection when the phenotype remains discordant.

The case may also illustrate diagnostic anchoring. A positive test can close a diagnostic pathway even when important features—here, marked splenomegaly, pancytopenia and ongoing fever—remain unexplained. Structured diagnostic reconsideration may help reduce the risk of diagnostic anchoring [13].

## Conclusion

In patients with suspected lymphoma, confirmed tuberculosis and persistent fever, splenomegaly, or cytopenias, concurrent visceral leishmaniasis should remain in the differential diagnosis when epidemiological and morphological clues support it [10]. Retrospective review of stored marrow or other tissue, combined with specialist Leishmania PCR, can reveal a second treatable infection and may help avoid unnecessary escalation when malignancy is under consideration.

## CRediT authorship contribution statement

**Nahid Ahmed:** Writing – original draft. **Justin Low:** Writing – review & editing. **Richard Hinton:** Writing – review & editing. **Brendan O’Kelly:** Writing – review & editing, Supervision.

## Consent for publication

Documented informed consent for publication of this de-identified case report and associated images was obtained from the patient.

## Ethics approval

Institutional ethics approval was not required for this single-patient case report under local policy.

## Declaration of Generative AI and AI-assisted technologies in the writing process

Generative AI tools were used for language editing, document restructuring and formatting assistance. The authors reviewed and approved the clinical facts, interpretation, authorship, citations, and final wording and retain responsibility for the manuscript.

## Funding

No specific funding was received.

## Declaration of Competing Interest

The authors declare that they have no known competing financial interests or personal relationships that could have appeared to influence the work reported in this paper.

## Data Availability

All relevant de-identified clinical data are included. Additional information is not publicly available because of patient confidentiality.
